# HIV-1 Hijacking of Host ATPases and GTPases That Control Protein Trafficking

**DOI:** 10.3389/fcell.2021.622610

**Published:** 2021-07-08

**Authors:** Lucas A. Tavares, Yunan C. Januário, Luis L. P. daSilva

**Affiliations:** Department of Cell and Molecular Biology, Center for Virology Research, Ribeirão Preto Medical School, University of São Paulo, Ribeirão Preto, Brazil

**Keywords:** HIV-1, GTPases, ATPases, HIV-1 accessory proteins, HIV-1 pathogenesis, HIV-1 trafficking

## Abstract

The human immunodeficiency virus (HIV-1) modifies the host cell environment to ensure efficient and sustained viral replication. Key to these processes is the capacity of the virus to hijack ATPases, GTPases and the associated proteins that control intracellular protein trafficking. The functions of these energy-harnessing enzymes can be seized by HIV-1 to allow the intracellular transport of viral components within the host cell or to change the subcellular distribution of antiviral factors, leading to immune evasion. Here, we summarize how energy-related proteins deviate from their normal functions in host protein trafficking to aid the virus in different phases of its replicative cycle. Recent discoveries regarding the interplay among HIV-1 and host ATPases and GTPases may shed light on potential targets for pharmacological intervention.

## Introduction

The human immunodeficiency virus type 1 (HIV-1) is the etiologic agent of acquired immunodeficiency syndrome (AIDS) and the cause of one of the longest and most devastating viral pandemics in human history. Although highly active antiretroviral therapy (HAART) inhibits the spread of HIV-1, the currently available treatments do not eradicate the virus from infected individuals, and viral mutations may confer resistance to the available drugs. Therefore, research into additional therapeutic strategies against HIV-1 is of high importance. In almost 40 years of intensive study, much has been learned about how HIV-1 manipulates the molecular machinery of the host cell to its own benefit. HIV-1 hijacks many host proteins to ensure an efficient replication cycle and to evade the immune response, leading to pathogenesis.

**FIGURE 1:**
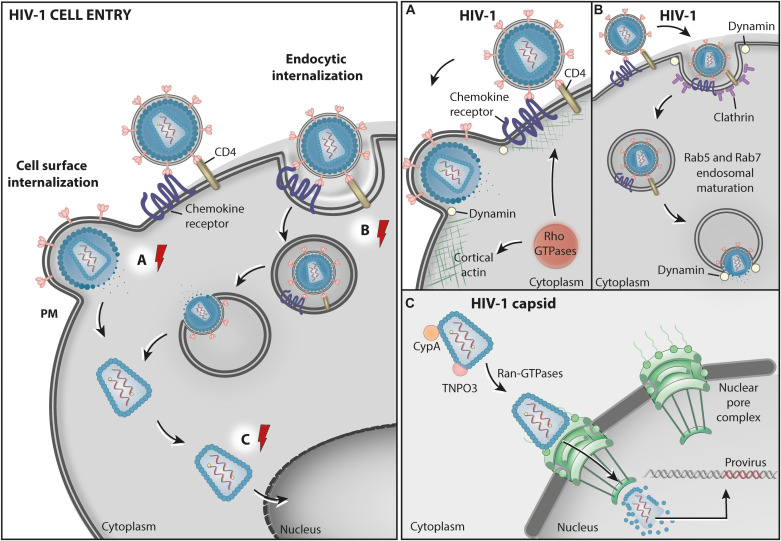
HIV1 cell entry. Schematic representation of two proposed pathways for HIV-1 entry into cells. The cell surface internalization pathway involves the fusion between the viral envelope and the cell PM and is believed to be the main route for HIV entry into permissive CD4 expressing cells **(A)** – Rho GTPases: RhoA, Rac1, and Cdc42 participate in HIV-1 entry by promoting fusion complex stabilization, and fusion pore formation and expansion at the host cell surface through actin cytoskeleton remodeling. The HIV-1 entry pore is also stabilized by Dynamin GTPase activity, facilitating the release of the capsid containing the virus genome into the cytosol. In an alternative, poorly characterized, endocytic internalization entry pathway, HIV-1 particles are endocytosed via clathrin-coated vesicles (CCVs) and delivered to endosomes. **(B)** Dynamin GTPase activity is involved in both the formation of CCVs and the fusion between the virus and the endosome membrane. This pathway also involves Rab5 and Rab7 acting in endosomal maturation. **(C)** The viral capsid in the cytosol is transported to the nuclear pore by CypA, TNPO3, and the nuclear transporter GTPase Ran-GDP while the RNA is reverse transcribed into cDNA. At the nucleoplasm, the HIV-1 provirus is integrated into the host chromatin. The red electric ray symbols represent critical steps in the transport of viral factors that require energy.

**FIGURE 2:**
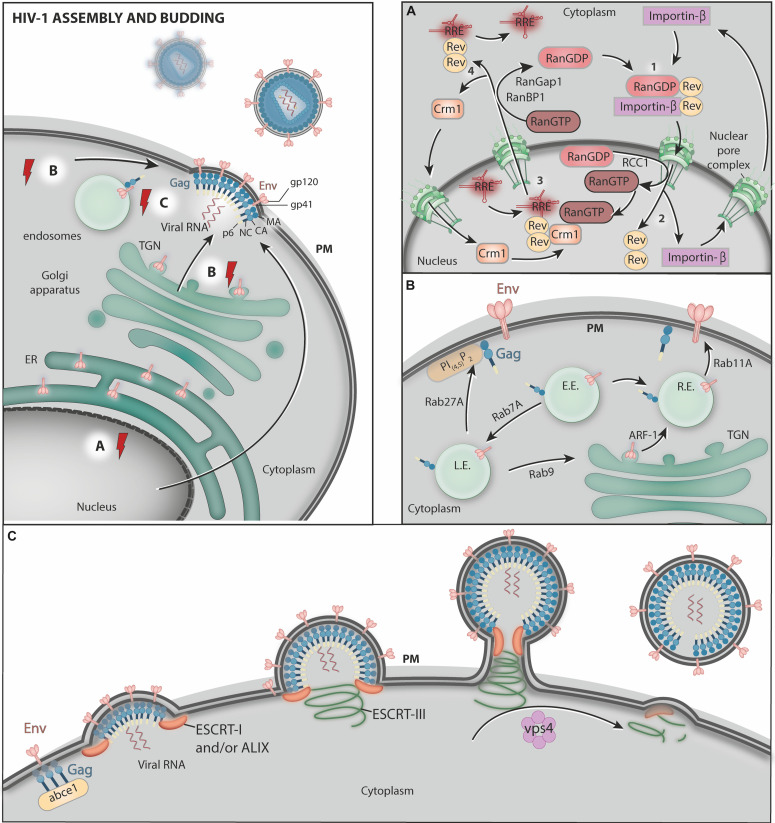
HIV-1 assembly and budding. **(A)** The host transcriptional machinery transcribes the viral genes. After synthesis in the cytoplasm, Rev associates with RanGDP and Importin-β, forming the complex Rev-Importin-β-RanGDP which is imported into nucleus through nuclear pores (step 1). In the nucleus, the conversion of RanGDP to RanGTP mediated by RRC1 disassemble Rev from Importin-β, allowing Importin-β to be exported back to the cytoplasm (step 2). Rev then binds RRE (present in viral RNA molecules), RanGTP, and Crm1 and the Crm1-RanGTP-Rev-RRE complex exits the nucleus through the nuclear pores (step 3). After the translocation to the cytoplasm, RanGTP is converted to RanGDP by RanGAP1 and RanBP1, allowing the disassembly of the complex, and Crm1 is imported again to the nucleus while transported viral RNAs are free to be translated (step 4). **(B)** Env is synthesized in association with the ER membrane and transported to the Golgi complex. After reaching the Golgi, Env is transported to the PM, where new HIV-1 particles are formed. This trafficking occurs via transport vesicles that require ARF-1 GTPase. Rab GTPases control several steps of HIV-1 structural protein trafficking in endosomes. Rab7A is required for mature Env incorporation into nascent virus particles. Rab9 was proposed to Env and Gag from the endolysosomal pathway back to the Golgi complex. This process reroutes HIV-1 proteins to virus assembly sites at the PM. Rab11 controls the pathway that recycles Env from the cell surface to endosomes and back to the PM. Rab27a activity helps to target Gag to virus assembly sites at the PM. Moreover, Rab8 and Rab7L1 (Rab29) activity are exploited by HIV-1 during *trans*-infection from dendritic cells (DCs) to target T cells via virological synapses (not shown). L.E., late endosomes; E.E., early endosomes; R.E., recycling endosomes. **(C)** Finally, ABCE1 facilitates Gag organization at the cell surface, where the ESCRT machinery and the AAA-ATPase VPS4 facilitate virus particle release. The red electric ray symbols represent critical steps in the transport of viral factors that require energy.

The HIV-1 replicative cycle in a host cell can be divided into early and late phases. The early phase (Figure 1) extends from virus entry to the integration of the provirus into the host cell genome and includes events such as the uncoating of the viral capsid, the reverse transcription of viral RNA to cDNA, the formation of the preintegration complex (PIC) and the nuclear import of the PIC. Although the order of these events is under debate (previously reviewed in Toccafondi et al., 2021). The late phase (Figure 2) starts with the transcription of the viral RNAs and comprises their subsequent nuclear export to the cytoplasm, the translation of the viral proteins, the trafficking of structural proteins to virus assembly sites, and the assembly, budding and maturation of the viral particle (previously reviewed in Freed, 2015).

The efficient transport of virus-derived proteins and nucleic acids to and from specific membrane-bound compartments within the host cell is critical in several steps of the HIV-1 replicative cycle. These translocation processes require specific transport and membrane remodeling machinery and a considerable amount of chemical energy provided by the host cell through ATP and GTP hydrolysis. Therefore, it is not surprising that HIV-1 co-opts several ATPases, GTPases and their regulators and effectors during infection and that these proteins are essential to virus replication and pathogenesis. Especially relevant among the GTPases are the small GTPases of the Ras superfamily, which are known to control critical processes implicated in intracellular trafficking. These include actin network dynamics, membrane specification, transport vesicle formation, translocation across the cytosol, and tethering to acceptor membranes. These monomeric proteins are found in GDP- or GTP-bound forms switching between inactive and active states in a cycle controlled by GAPs (GTPase-activating proteins) and GEFs (nucleotide exchange factors), respectively (previously reviewed in Itzen and Goody, 2011). When in their GTP-bound active state, these GTPases are membrane-associated and may interact with specific effector molecules.

This review presents examples and discusses data regarding the interplay between HIV-1 and host ATPases and GTPases (Table 1) involved in the intracellular trafficking of macromolecules and membrane modification. We will also discuss cases in which the subcellular localization of transmembrane ATPases themselves is altered by HIV-1 to aid virus replication and spread. Host restriction factors with ATPase and GTPase activity acting against HIV will not be covered here, and we refer to recent reviews (Ghimire et al., 2018; Staeheli and Haller, 2018).

**TABLE 1:** ATPases and GTPases co-opted by HIV-1 during its replication cycle are presented in the order that they appear in the text.

ATPase/GTPase	Process in the HIV-1 replicative cycle	References
Dynamin	• Viral membrane fusion with the host cell. • Stabilization of the HIV-1 entry pore. • Work together with Actin and Bar domain proteins to facilitate the release of viral nucleocapsid into the cytosol.	Miyauchi et al., 2009; de la Vega et al., 2011; Taylor et al., 2012; Aggarwal et al., 2017; Jones et al., 2017
Rab5 and Rab7	• Involved in endocytic entry of HIV-1	Vidricaire and Tremblay, 2005; Marin et al., 2019
Ran	• HIV PIC nuclear import. • Interacts with TRN-SR2 to release HIV PIC in the nucleoplasm. • Nuclear egress of Crm1-Rev-RRE-cargo complex. • Ran GDP, together with Importin-β, promotes Rev nuclear import.	Malim et al., 1989a; Fornerod et al., 1997; Henderson and Percipalle, 1997; Neville et al., 1997; Askjaer et al., 1998; Christ et al., 2008; König et al., 2008; Dong et al., 2009; Monecke et al., 2009; Güttler et al., 2010; Krishnan et al., 2010; De Iaco and Luban, 2011; Zhou et al., 2011; Larue et al., 2012; Valle-Casuso et al., 2012; Taltynov et al., 2013; Behrens et al., 2017
ABCE1	• HIV-1 assembly.	Lingappa et al., 1997, 2006; Zimmerman et al., 2002; Dooher and Lingappa, 2004; Dooher et al., 2007; Klein et al., 2011; Reed et al., 2018
ARF-1	• Viral particle production. • *Trans*-infection at virological synapse.	Joshi et al., 2008, 2009b; Bayliss et al., 2020
ARF-3	• Viral particle production.	Joshi et al., 2008
ARF-5	• Viral particle production.	Joshi et al., 2008
ARF-6	• Regulates CD4-dependent HIV-1 entry and infection by controlling PI(4,5)P2 dynamics at PM.	García-Expósito et al., 2011
Rab7A	• Env processing. • Env incorporation into virions. • Release of the viral particle.	Caillet et al., 2011
Rab7L1 (Rab29)	• *Trans*-infection at virological synapse.	Bayliss et al., 2020
Rab8A	• *Trans*-infection at virological synapse.	Bayliss et al., 2020
Rab9	• Viral particle production. • Gag transport to HIV-1 assembly site. • Together with TIP47, help the retrograde transport of Env from endosome to TGN.	Blot et al., 2003; Murray et al., 2005
Rab11A	• Control the levels of cellular and viral particle-associated Env. • FIP1C (Rab11 effector) reroutes Env to recycling endosomes.	Qi et al., 2013; Kirschman et al., 2018
Rab27A	• Participates in Gag, PI4KIIα and PI_(__4_,_5__)_P_2_ levels at cell surface. • HIV-1 assembly. • *Trans*-infection at virological synapse.	Gerber et al., 2015
Rac1	• Promote pore formation and expansion during HIV-1 entry • control Gag levels at the cell surface and VLP release. • Gag expression activates Rac1 and increases F-actin content.	Pontow et al., 2004, 2007; Harmon et al., 2010; Vorster et al., 2011; Thomas et al., 2015
RhoA	• HIV-1 entry via stabilization of the fusion complex. • gp41 binds p115-RhoGEF which play a role in viral replication. • Control monocyte migration and adhesion, which may affect HIV-1 dissemination. • Activation of a RhoA–ROCK–LIMK–Cofilin signaling cascade mediated by Filamin-A facilitates HIV-1 entry	Zhang et al., 1999; Wang L. et al., 2000; Hodges et al., 2007; Jiménez-Baranda et al., 2007; Lucera et al., 2017
Cdc42	• Promotes plasma membrane expansions that facilitate viral transfer from DCs to T cells • Promotes HIV-1 T cell infection.	Nikolic et al., 2011; Shrivastava et al., 2015; Lucera et al., 2017
VPS4	• ESCRT-dependent HIV-1 assembly and budding.	Baumgärtel et al., 2011; Jouvenet et al., 2011; Bleck et al., 2014; Van Engelenburg et al., 2014; Johnson et al., 2018

## HIV-1 Cell Entry

HIV-1 enters cells mainly by fusing its envelope membrane to the plasma membrane (PM) of target cells (Figure 1). This process requires physical interactions between the gp120 subunit of the virus envelope (Env) protein and specific host proteins at the cell surface that function as the main receptor (CD4) (Maddon et al., 1986; Mcdougal et al., 1986) and as coreceptors (either CXCR4 or CCR5) (Alkhatib et al., 1996; Dragic et al., 1996; Feng et al., 1996) for the virus. These interactions expose a fusion domain in the gp41 subunit of Env, leading to membrane fusion and the delivery of the capsid containing the viral genome into the cytoplasm (Wilen et al., 2012).

There is also evidence, obtained mainly from non-canonical HIV-1 target cells, indicating that the virus may enter cells via endocytosis (Fackler and Peterlin, 2000; Fredericksen et al., 2002; Daecke et al., 2005). Consistent with this model, the impairment of endosome acidification by drug treatments was shown to compromise the infection of polarized trophoblasts with HIV, suggesting that incoming viruses are delivered to endosomes, where the acidic pH would facilitate membrane fusion (Vidricaire and Tremblay, 2005). In fact, the expression of small GTPases Rab5 or Rab7 mutants, known to impair endosome maturation, also inhibited HIV-1 infection in those cells (Vidricaire and Tremblay, 2005). Recently, Marin et al. (2019) found that the knockdown of Rab5, Rab11A or the Rab effector protein RABEP1 decrease HIV-1 fusion with endosomes in CD4+ T cells, highlighting the importance of the endocytic machinery in the HIV-1 entry process.

Regardless of the exact pathway, evidence from independent studies indicates that the GTPase Dynamin participates in the HIV-1 entry process (Daecke et al., 2005; Miyauchi et al., 2009; de la Vega et al., 2011; Jones et al., 2017). Dynamin is a GTPase best known for catalyzing membrane fission during clathrin-mediated endocytosis (CME). Based on this well-characterized role, it was originally proposed that Dynamin is involved in HIV-1 entry by endocytosis, possibly via CME (Daecke et al., 2005; Miyauchi et al., 2009). The authors of this study showed that pharmacological inhibition of Dynamin or overexpression of a Dynamin dominant-negative (K44) mutant impaired HIV-1 infection (Miyauchi et al., 2009). However, the exact molecular connection between HIV entry and Dynamin was not elucidated.

A study by de la Vega and collaborators provided a hint on the mechanism by which Dynamin assists on HIV entry. They showed that Dynamin activity, which was inhibited with the drug dynasore, is required for the efficient fusion of the virus with target cell membranes and the release of the viral content into the cytosol (de la Vega et al., 2011). The authors proposed that in addition to playing a role in HIV-1 endocytosis, Dynamin may also facilitate virus fusion with the endosome membrane. Importantly, under dynasore treatment virus fusion with the PM was also impaired (de la Vega et al., 2011), most likely also contributing to inhibit viral entry in the experimental setup. Indeed, new data show that Dynamin participates in HIV-1 entry via a CME-independent mechanism (Aggarwal et al., 2017; Jones et al., 2017). In this process, Dynamin molecules associate to form tetramers that stabilize the HIV entry pore, facilitating the release of viral nucleocapsids into the cytosol through the actin cortex at the cell surface (Jones et al., 2017). The authors proposed that Dynamin may indirectly control this process by recruiting effector proteins. In support of this model, a previous report from Taylor et al. (2012) revealed that Dynamin proteins work cooperatively with actin and N-terminal containing BAR (BIN/Amphiphysin/RVS) domain proteins at sites of membrane remodeling at the cell surface. The roles of actin cytoskeleton dynamics in HIV entry will be discussed later in this review (see section “Rho GTPases”).

Finally, the fluidity of the PM, regulated by the presence of the lipid phosphatidylinositol (4,5)-bisphosphate [PI(4,5)P2], was shown to be crucial for HIV-1 entry in lymphocytes (Barrero-Villar et al., 2008). The small GTPase ADP ribosylation factor 6 (ARF-6) plays a significant role in this process by regulating the PI(4,5)P2 enrichment at the inner leaflet of the PM. García-Expósito et al. (2011) showed that expression of ARF-6 mutants defective on GTP/GDP cycle caused the accumulation of PI(4,5)P2-associated structures at the cell surface impeding CD4-dependent HIV-1 entry and infection, but without affecting CD4-viral attachment (García-Expósito et al., 2011). These results indicate that efficient early HIV-1 infection of permissive cells requires ARF6-mediated PM dynamics, possibly affecting pore formation.

## HIV-1 Transport to the Nucleus and Translocation to the Nucleoplasm

The fusion of the HIV-1 envelope with the target cell membrane results in the delivery of the viral capsid containing the viral genomic RNA (gRNA) into the cytoplasm (Figure 1). The HIV-1 capsid core comprises a conical structure made of capsid (CA) protein, which contains the gRNA and copies of the viral reverse transcriptase and integrase enzymes. In addition to protecting the viral genome from host restriction factors and innate immunity recognition, the capsid provides an optimized environment for reverse transcription, leading to the synthesis of viral DNA that is, ultimately, integrated into the host cell chromatin to form a provirus (Figure 1).

Viral capsid uncoating/disassembly is required for integration and productive infection. However, the timing and location of capsid disassembly is still a matter of debate, with models proposing that it occurs either at the cytoplasm, at the nuclear pore, or within the nucleus (previously reviewed in Toccafondi et al., 2021). Several recent studies favor a model in which capsid disassembly occurs after complete nuclear translocation by providing evidence that reverse transcription finalizes within the nucleus and that the capsids enter the nucleus and remain intact (or nearly intact) until they uncoat near the integration sites, just minutes before integration (Burdick et al., 2020; Dharan et al., 2020; Selyutina et al., 2020; Li et al., 2021). The recent direct visualization of apparently intact, cone-shaped HIV-1 capsids being imported through nuclear pores in infected T cells provides definite proof that capsid uncoating may occur after nuclear transport is complete (Zila et al., 2021).

The nuclear pore complexes and the soluble transport receptors of the karyopherin family of proteins mediate the transport of macromolecules across the nuclear envelope. This process requires an input of energy derived from GTP hydrolysis by Ras-related nuclear (Ran) small GTPases, which provide selectivity and directionality to the nuclear translocation process. Interaction between GTP-bound Ran (Ran-GTP) and nuclear transport receptors directs the binding and release of cargo, and the enrichment of Ran-GDP in the cytoplasm and Ran-GTP in the nucleoplasm forms a gradient that controls the bidirectional flow of molecules in and out of the nucleus (Koyama and Matsuura, 2010).

Several components of the nuclear transport machinery are required for HIV infection, including the β-karyopherin Transportin-SR2 (TRN-SR2, also known as TNPO3) (Brass et al., 2008; Christ et al., 2008; König et al., 2008). Consistent with a model in which HIV-1 hijacks the Ran-GTPase system to invade the nucleus, Taltynov et al. (2013) observed that TNPO3 associates with Ran-GTP, which may facilitate the release of viral material in the nucleoplasm. TNPO3 requirement during HIV-1 and other lentivirus infection was correlated to capsid binding and proposed to occur after nuclear entry (Krishnan et al., 2010; De Iaco and Luban, 2011; Valle-Casuso et al., 2012). Interestingly, recombinant TNPO3 stimulates the uncoating of HIV-1 cores *in vitro*, a property that is inhibited by the CA-binding host protein cyclophilin A (CypA) (Shah et al., 2013). This relationship between TNPO3 and CypA suggests that these proteins coordinate proper disassembly of the viral capsid in target cells, with TNPO3 favoring capsid disassembly within the nucleus.

Although TNPO3 was shown initially to bind integrase (Christ et al., 2008), the role of this interaction in HIV-1 infection is presently not clear (Cribier et al., 2011) and, in light of new data discussed above, may occur within the nucleus. Nevertheless, a library with more than 25,000 small molecules was recently screened for inhibitors of the HIV-1 integrase-TNPO3 interaction, and new compounds that significantly reduce HIV-1 integration were identified (Demeulemeester et al., 2018). These compounds may represent potential future drugs to treat HIV infection. Despite these advances, much remains to be learned about the process by which HIV-1 material is imported into the nucleus, as other host factors are likely to be required for this process. The emerging candidates include importin α, importin β, and importin 7, but divergent data indicate a complex process, and other proteins may also be involved (previously reviewed in Matreyek and Engelman, 2013).

## Nuclear Exit of HIV Products

The late phase of the HIV-1 replication cycle starts with provirus gene expression (Figure 2). The transcription of the HIV-1 genome is mediated by host cell RNA polymerase II and initiates from the U3 promoter region within the proviral 5′LTR. To achieve maximum production, this process is enhanced by the viral regulatory protein Tat (transactivator of transcription) (Siekevitz et al., 1987). HIV-1 RNA synthesis and downstream processing result in the production of a variety of RNA species, including completely spliced mRNA molecules [encoding the Tat, regulator of expression of virion proteins (Rev), and negative factor (Nef) proteins], partially spliced mRNAs (encoding the structural proteins Gag, Pol and Env), and unspliced gRNA (previously reviewed in Cullen, 2003), which need to reach the cytosol for protein synthesis and/or virion assembly.

HIV-1 RNA export from the nucleus is mainly mediated by the regulatory protein Rev, which facilitates the nuclear export and cytoplasmic build-up of singly spliced and unspliced viral RNA molecules (Malim et al., 1989b) through an energy-consuming process. Rev binds and oligomerizes to Rev response elements (RREs) present in viral RNA molecules, allowing the formation of ribonucleoproteins that are competent for nuclear export. This process evades the host cell quality control mechanisms that prevent the nuclear export of incompletely spliced RNA molecules (Malim et al., 1989a). The Rev–RRE ribonucleoprotein complex interacts with host export factor Crm1, an karyopherin family member, also known as exportin 1 (Xpo1) (Fornerod et al., 1997; Güttler et al., 2010).

The nuclear egress of the Crm1-Rev-RRE-cargo complex through nuclear pores is an energy-dependent process. This event is controlled by Ran GTPase, which enables the formation of the Crm1-RanGTP-Rev-RRE-cargo complex. In fact, Ran in its active (GTP-bound) form mediates the interaction between Crm1 and Rev through a surface-exposed hydrophobic pocket on Crm1 (Dong et al., 2009; Monecke et al., 2009; Güttler et al., 2010). Recently, Behrens et al. (2017) have shown that Rev tolerates several nuclear export signals, even those that bind Crm1 in a Ran-GTP-independent manner. Additionally, interactions between Rev proteins may mask their nuclear export signals and favor the nuclear accumulation of Rev (Behrens et al., 2017).

Once in the cytoplasm, the Crm1-RanGTP-Rev-RRE-cargo complex is dissociated as a consequence of GTP hydrolysis induced by RanGAP1 and RanBP1, which releases Cmr1 and RanGDP from the Rev-RRE-cargo complex (Neville et al., 1997; Askjaer et al., 1998). Thereafter, the RRE disassociates from Rev and the RRE-RNA is translated, whereas Crm1 is re-imported into the nucleus. In the cytoplasm, an importin-β binds to the nuclear localization signal of Rev and, combined with RanGDP, promotes the nuclear import of Rev via the nuclear pores (previously reviewed in Meyer and Malim, 1994; Truant and Cullen, 1999; Suhasini and Reddy, 2009). In the nucleus, the disassembly of the Rev-RanGDP-Importin-β complex is triggered by the conversion of RanGDP to RanGTP, resulting in the release of Rev to facilitate further viral RNA nuclear export (previously reviewed in Meyer and Malim, 1994; Henderson and Percipalle, 1997; Suhasini and Reddy, 2009).

## HIV-1 Assembly and Budding

The HIV-1 structural proteins Gag and Env are synthesized in the cytosol and in association with the endoplasmic reticulum (ER) membrane, respectively, and their correct targeting to viral assembly sites is crucial in HIV-1 replication (Figure 2). Although Gag protein is sufficient for the assembly and budding of virus-like particles (VLPs) (Gheysen et al., 1989) as well as for the recruitment and packaging of the viral genome into VLPs (Shields et al., 1978; Sakalian et al., 1994), the proper incorporation of Env glycoproteins into the nascent virion is essential to the production of infectious particles. Depending on the cell type, HIV-1 assembly has been proposed to take place at the inner leaflet of the PM (Ono et al., 2000; Ono and Freed, 2004), at the intraluminal vesicles (ILVs) of late endosomes/multivesicular bodies (MVBs) (Nydegger et al., 2003; Sherer et al., 2003; Joshi et al., 2009a) or at intracellular compartments connected to the cell surface by tubules (Bennett et al., 2009). However, how Env and Gag reach the sites of viral assembly in each case is not fully understood. Intense study on this subject has revealed a number of host ATPases and GTPases involved in HIV-1 particle morphogenesis and budding.

### Small GTPases Regulating the Intracellular Trafficking and Subcellular Distribution of HIV-1 Structural Proteins

#### ARF GTPases

The ADP-ribosylation factors (ARFs) form a protein family within the Ras superfamily of small GTPases (previously reviewed in D’Souza-Schorey and Chavrier, 2006) and play important roles in intracellular vesicle trafficking, actin remodeling and phospholipid metabolism (previously reviewed in Kahn et al., 2005; D’Souza-Schorey and Chavrier, 2006; Gillingham and Munro, 2007; Randazzo et al., 2007; Kahn, 2009). In the context of HIV-1 infection, cellular depletion of ARF-1, ARF-3 or ARF-5 levels by RNAi or the expression of dominant-active forms of these molecules was shown to impair HIV-1 particle production (Joshi et al., 2008).

ARF molecules may initiate the formation of transport vesicles by recruiting vesicle coat components to specific donor membranes. Among the vesicle coat proteins recruited by ARFs are the monomeric Golgi-localized, γ-ear containing, ARF-binding (GGA) adaptors (Boman et al., 2000; Dell’Angelica et al., 2000) and members of the adaptor protein (AP) complex family (Stamnes and Rothman, 1993; Traub et al., 1993). GGAs and APs are sorting adaptors involved in protein trafficking in the late secretory pathway and have been implicated in the intracellular transport of Gag and Env. Specifically, GGA1-3 (Joshi et al., 2008, 2009b), AP-1 (Camus et al., 2007), AP-2 (Batonick et al., 2005), AP-3 (Dong et al., 2005; Garcia et al., 2008; Alford et al., 2016), and AP-5 (Alford et al., 2016) have been described to play key roles in the correct targeting of Gag to viral assembly sites. GGA overexpression was shown to compromise viral particle production, and mutation of the ARF-binding sites in GGAs abrogated this phenotype (Joshi et al., 2008, 2009b). The authors of this study suggested that GGA overexpression hinders viral production by sequestering free ARF-1 molecules and thus impairing its activity.

The HIV-1 Env polyprotein precursor gp160 is processed in the Golgi complex to produce gp120 (surface glycoprotein) and gp41 (hydrophobic transmembrane glycoprotein), which are bound to each other in a non-covalent manner (McCune et al., 1988; Freed et al., 1989; Stein and Engleman, 1990; Hallenberger et al., 1992). Importantly, gp160 processing is essential for efficient HIV-1 membrane fusion and infectivity (McCune et al., 1988; Freed et al., 1989; Bosch and Pawlita, 1990; Guo et al., 1990; Dubay et al., 1995). From the Golgi, gp120-gp41 heterodimers are transported to the cell surface, possibly via endosomes, in an ARF-1/AP-1-dependent manner (Berlioz-Torrent et al., 1999; Wyss et al., 2001).

Recently, an RNAi-based screen of membrane trafficking regulators showed that ARF-1 depletion in dendritic cells (DCs) reduced the transfer of viral particles to T cells by rerouting Gag molecules away from the sites of virological synapsis, leading to virus particles accumulation in small vesicle structures at the donor cell periphery (Bayliss et al., 2020). However, this inhibitory effect in virus transfer may not be merely due to altered Gag trafficking, because ARF-1 depletion also affected the delivery of virological synapse (VSs) structural proteins to the PM, such as the tetraspanin CD81 (Bayliss et al., 2020). Therefore, it is not currently possible to conclude whether ARF-1 plays a direct role in Gag trafficking and HIV-1 cell-to-cell transfer.

#### Rab GTPases

The Rab (Ras-related in brain) proteins form another family of small GTPases involved in the trafficking of HIV-1 Gag and Env. In general, Rabs provide identity and function to the membranes of secretory pathway compartments with multiple functions in vesicle transport (previously reviewed in Lamber et al., 2019; Homma et al., 2021). The roles of Rab proteins in viral replication have been recently reviewed (Spearman, 2018). Regarding HIV, a study by Caillet et al. (2011) implicated the activity of several Rab proteins (Rab4A, Rab6A, Rab7A, Rab8A, Rab9A, and Rab11A) in viral replication. Among these Rabs, the function of Rab7A (an endosome-associated Rab) was described as being the most important for efficient HIV-1 particle production (Caillet et al., 2011). The authors showed that the depletion of Rab7A impairs both Env processing and the incorporation of mature Env into viral particles, compromising viral infectivity. Moreover, Rab7A depletion hampers the release of HIV-1 progeny, which accumulates at the cell surface (Caillet et al., 2011). Interestingly, this phenotype was dependent on the expression of the host restriction factor BST2/Tetherin, which is normally antagonized by the HIV-1 accessory protein viral protein U (Vpu) (Neil et al., 2008); this process is discussed later in this review (see section “Vpu”). Furthermore, a recent study showed that trafficking pathways controlled by Rab8A and Rab7L1 (also known as Rab29) are exploited by HIV-1 during *trans*-infection from DCs to target T cells via VSs. The authors showed that the depletion of Rab8A and Rab29 in DCs leads to a reduction in HIV-1 localization at VSs and a consequent accumulation of Gag and CD81, a host tetraspanin that is normally recruited to VSs, in intracellular compartments (Bayliss et al., 2020).

Another Rab potentially involved in HIV-1 replication is Rab9 (Murray et al., 2005). Rab9 was originally linked to protein transport between endosomes and the TGN (Lombardi et al., 1993; Shapiro et al., 1993; Riederer et al., 1994) and more recently to lysosomes and lysosome-related organelles biogenesis and autophagy (Riederer et al., 1994; Ganley et al., 2004; Nishida et al., 2009; Kloer et al., 2010). Using gene-trap insertional mutagenesis and RNAi assays, it was found that interfering with Rab9 activity causes the rerouting of Gag to lysosomes and a decrease in HIV-1 particle production (Murray et al., 2005). Interestingly, TIP47 (also known as Perlipin-3), a Rab9 interacting protein originally implicated in protein trafficking (Díaz and Pfeffer, 1998; Hanna et al., 2002) and more recently in lipid droplet biogenesis (Wolins et al., 2001; Bulankina et al., 2009), was proposed to play a role in HIV-1 particle biogenesis (Blot et al., 2003; Lopez-Vergès et al., 2006). A study showed that TIP47/Perlipin-3 binds Env via a di-aromatic Y_802_W_803_ motif in gp41 that is required for proper retrograde transport of Env from endosomes to the TGN and for Env incorporation into virions (Blot et al., 2003). TIP47 also interacts with Gag via the matrix (MA) domain, and a study show that Env incorporation was inhibited by TIP47 depletion or by the disruption of the Gag-TIP47 interaction (Lopez-Vergès et al., 2006). The authors proposed that TIP47 may function as a connector between Env and Gag, controlling proper Env incorporation during viral particle assembly. However, this notion was challenged by a more recent study (Checkley et al., 2013). Although TIP47 interaction with MA was confirmed, the authors of this other study did not observe changes in Env incorporation, virus release, infectivity, or replication upon TIP47 depletion in either HeLa cells or Jurkat T cell lines. Therefore, the mechanism by which Rab9 and TIP47 affect HIV-1 replication remains unclear.

Strong evidence for the ability of HIV-1 proteins to co-opt Rabs and Rab effectors in recycling endosome-mediated pathways has also come to light. Rab11 is one of the main regulators of membrane recycling in the late secretory pathway (Ullrich et al., 1996; Casanova et al., 1999; Wang X. et al., 2000; Hales et al., 2001). Although Rab11A depletion does not alter Env incorporation into virions, the expression of a Rab11A active (GTP-bound) form decreases the levels of cellular and particle-associated Env (Qi et al., 2013). Consistent with this result, Env expression modifies the localization of the Rab11 effector FIP1C from recycling endosomes to the cell periphery, and FIP1C depletion reduces the levels of Env in cells and nascent viral particles, delaying HIV-1 replication (Qi et al., 2013). Furthermore, the expression of a C-terminal fragment of FIP1C (FIP1C_560__–__649_) reroutes Env to recycling endosomes and diminishes the levels of Env on the cell surface and the incorporation of Env into virions (Kirschman et al., 2018). The function of Rab11/FIP1C may also involve Rab14 because the expression of a FIP1C mutant (S_580_N/S_582_L) that does not bind Rab14 similarly depletes Env from the viral particles (Qi et al., 2013), a phenotype recapitulated by the expression of an inactive GDP-bound Rab14 (Rab14S_25_N) (Qi et al., 2013).

Importantly, it is known that the lipid phosphatidylinositol (4,5)-bisphosphate [PI(4,5)P2] present in the inner leaflet of the PM plays an essential role in Gag localization at the PM (Ono et al., 2000; Ono and Freed, 2004). In a process called the “myristoyl switch,” the binding of the negatively charged inositol headgroup of PI(4,5)P2 to the MA domain of Gag exposes the N-terminal myristoyl group present in the MA domain, which mediates the anchorage and stabilization of Gag at the PM (Ono et al., 2000; Ono and Freed, 2004; Brügger et al., 2006; Saad et al., 2006; Shkriabai et al., 2006). Additionally, Gag traps PI(4,5)P2 and cholesterol, suppressing their mobility at the T cell PM and creating an efficient microdomain platform for virus assembly (Favard et al., 2019). Taking these findings into consideration, Gerber et al. (2015) carried out a study that revealed that Rab27A controlled PI(4,5)P2 levels at HIV-1 assembly sites by directing the enzyme PI4KIIα (phosphatidylinositol 4-kinase type 2 α) from late endosomes to the PM, where PI4KIIα produces phosphatidylinositol 4-phosphate [PI(4)P], a precursor of PI(4,5)P2 (Rameh et al., 1997; Doughman et al., 2003). Therefore, the depletion of Rab27A reduced the pools of PI4KIIα and PI(4,5)P2 at the cell surface, reducing Gag association with the PM and HIV-1 assembly/production (Gerber et al., 2015). Although the trafficking and processing of Env were not affected in Rab27A-silenced cells, Rab27A ablation impaired HIV-1 cell-to-cell spread either through free viral particles or by *trans*-infection at the VSs (Gerber et al., 2015). Finally, expression of the Rab27A effector proteins Slac2b, Slp2a, and Slp3 was also shown to be required for Gag association with the PM and efficient HIV-1 particle production (Gerber et al., 2015). Altogether, these results demonstrate an important role for Rab27A and its effector proteins in HIV-1 production and spread through a PI4KIIα trafficking mechanism.

#### Rho GTPases

The Rho GTPase family (including Cdc42, Rac1, and RhoA) regulates multiple cellular processes involving the activation of signaling pathways, such as cell adhesion, migration, survival, differentiation, and proliferation (Tybulewicz and Henderson, 2009). Given the intricate manipulation of host cell signaling by HIV-1, it is not surprising that several studies have reported that Rho GTPases are involved in various events in the HIV-1 replication cycle. Rho GTPases are especially relevant in the host actin cytoskeleton subversion by HIV-1, a recently reviewed subject (Stella and Turville, 2018). There is strong evidence that incoming HIV-1 particles interfere with actin cytoskeleton dynamics at the target cell cortex by triggering the activation of signaling cascades mediated by distinct Rho GTPases (Figure 1).

Jiménez-Baranda et al. (2007) showed that the interaction of Env with CD4 and the chemokine coreceptors (CCR5 and CXCR4) promote F-actin stabilization via phosphorylation (and inactivation) of Cofilin, an F-actin depolymerizing factor (Jiménez-Baranda et al., 2007). The LIM domain kinase 1 (LIMK1), a protein that phosphorylates Cofilin is activated by RhoA or Rac1 effectors ROCK and PAK-1, respectively (Maekawa et al., 1999; Vadlamudi et al., 2002; Jiménez-Baranda et al., 2007). Interestingly, Filamin-A, an actin cross-linking protein that binds ROCK and PAK-1 (Ohta et al., 2006), also interacts with CD4, CCR5, and CXCR4 (Jiménez-Baranda et al., 2007), and this interaction was shown to be required for Env-mediated RhoA activation, Cofilin phosphorylation, and efficient HIV-1 infection. Since HIV-1 infection was impaired by a ROCK inhibitor, and not by disruption of Rac1 or PAK-1 activity, it was proposed that Filamin-A mediates activation of a RhoA–ROCK–LIMK–Cofilin signaling cascade that facilitates HIV-1 entry via stabilization of the fusion complex (Jiménez-Baranda et al., 2007).

In contrast, work from other groups support a crucial role for Rac1 activation in promoting HIV infectivity. Vorster et al. (2011) showed that HIV-1 infection, or gp120 treatment, activates Rac1 and induces PAK-mediated activation of LIMK1, leading to phosphorylation and inactivation of Cofilin in resting CD4 T cells (Vorster et al., 2011). In fact, the interaction of Env with CD4 and CCR5 or CXCR4 was shown to trigger a defined Gαq-mediated signaling cascade activating Rac1 to promote actin polymerization events at the cell cortex that facilitates fusion (Pontow et al., 2004; Harmon and Ratner, 2008). Further studies into this mechanism show that this Env-induced signaling cascade activates the tyrosine kinase Abl that stimulates the Rac GEF Tiam-1 to activate Rac1. Subsequently, Abl and Rac1-GTP activate the Wave2 complex, which stimulates actin polymerization via the Arp2/3 complex (Harmon et al., 2010). Because pharmacological inhibition of Abl was shown to arrest Env-mediated membrane fusion at the hemifusion step, the authors of this study proposed that actin remodeling mediated by Abl and Rac1 promote pore formation and expansion during HIV-1 entry (Harmon et al., 2010).

Binding of Env to target cells was also shown to activate Cdc42 and contribute to viral spreading. Env-binding to the DC-SIGN protein in the surface of DCs, triggers Cdc42 activation via a c-Src cascade leading to filopodia formation via the Arp2/3 complex and Diaphanous 2. These PM expansions in DC cells where shown to facilitate viral transfer to T cells (Nikolic et al., 2011; Shrivastava et al., 2015). Moreover, DC-SIGN stimulation by HIV-1 in monocyte-derived DCs results in a complex formation containing DC-SIGN, Rho and a Rho GEF called leukemia-associated Rho guanine nucleotide exchange factor (Rho GEF) (LARG) (Hodges et al., 2007). The Rho activation mediated by LARG via DC-SIGN is essential for optimal HIV-1 replication and VS formation (Hodges et al., 2007). The extensive manipulation of Rho GTPases pathways during HIV entry was nicely illustrated in a recent study by Lucera et al. (2017). Using a phosphoproteomics approach, the authors confirmed that HIV binding to CD4 and CCR5 activates Rac1 and Cdc42 leading to dramatic changes in the phosphorylation status of proteins associates to GTPase signaling (Lucera et al., 2017).

While the importance of Rho GTPases activity in HIV entry and spreading seems to be a consensus in the field, the relative importance of the family members (either Cdc42, Rac1, or RhoA) in the specific process has been a matter of controversy. This difficulty is partially due to the great degree of overlap and crosstalk among the Rho GTPases signaling pathways with shared regulators and downstream effectors. For instance, the LIMK-Cofilin pathway unites all three major Rho GTPases. The increasing availability of specific inhibitors targeting the GTPases themselves will help clarify the individual functions of Rho GTPases in HIV replication processes and provide valuable tools to fight infection.

Besides their role in virus entry, there is a well-documented interplay between Rho GTPases and the HIV-1 structural proteins during viral assembly/replication. It has been demonstrated that the cytosolic tail of HIV-1 gp41 (gp41C) binds the regulatory domain of p115 (Zhang et al., 1999), a RhoA GEF (Hart et al., 1996). This interaction is functionally relevant since the disruption of the p115-RhoGEF binding site in gp41 (Zhang et al., 1999) or the overexpression of a p115-RhoGEF activator (Wang L. et al., 2000) were shown to inhibit HIV-1 replication. Moreover, gp41C expression inhibited p115-RhoGEF-mediated actin stress fiber formation (Zhang et al., 1999), suggesting that HIV-1 regulates RhoA activity via its GEF. Because RhoA activity was shown to normally regulate the cell survival (Galandrini et al., 1997) and migration (Aepfelbacher et al., 1996) of HIV-1 target cells, the gp41C-p115-RhoGEF interaction and RhoA activity are likely to affect HIV-1 dissemination and pathogenesis (Zhang et al., 1999; Wang L. et al., 2000).

Finally, the trafficking of Gag to HIV-1 assembly sites was shown to be dependent on Rac1, where the levels of Gag at the PM and the release of HIV-1 Gag VLPs were inhibited in Rac1-depleted T cells (Thomas et al., 2015). In fact, the efficient production of HIV-1 Gag VLPs requires a functional Rac1-Wave2-IRSp53-Arp2/3 pathway in T cells (Thomas et al., 2015). Although, the HIV-1 accessory protein Nef potently induces Rac1 activation (as discussed later in this review, see section “Nef”) and may contribute to the role of Rac1 in virus release, Gag expression alone activates Rac1 and increases intracellular F-actin content (Thomas et al., 2015).

### ATPases Involved in HIV Budding

#### The ATPAse ABCE1

The membrane budding process driven by the HIV-1 Gag protein requires ATP, given that it is inefficient in ATP-depleted cells (Lingappa et al., 1997; Tritel and Resh, 2001). Since HIV-1 Gag does not itself interact with ATP and it is unable to harness its stored chemical energy, researchers sought to identify a host protein that could serve as an adaptor in this process. Using coimmunoprecipitation assays, Lingappa’s lab identified the interaction of Gag with ABCE1 (also called HP68 or RNase L inhibitor) (Zimmerman et al., 2002), a member of subfamily E of the ATP-binding cassette (ABC) ATPases (Dean et al., 2001). The authors showed that ABCE1 interacts with capsid assembly intermediates and is essential for immature capsid formation in a cell-free system, a notion confirmed by expressing an ABCE1 dominant-negative protein in cells (Zimmerman et al., 2002). Because ABCE1 binding appears to promote conformational changes in nascent capsid structure (Zimmerman et al., 2002), this ATPase may act as a Gag chaperone during oligomerization to facilitate viral particle assembly. Indeed, the function of ABCE1 in the capsid assembly pathway is energy-dependent and occurs in a stepwise manner, involving its progressive association to assembly intermediates (Dooher and Lingappa, 2004). Moreover, the requirement of ABCE1 is conserved for several primate lentivirus capsids, such as HIV-1, HIV-2, and simian immunodeficiency viruses (SIV_*mac*__239_ and SIV_*agm*_) (Dooher and Lingappa, 2004) and non-primate lentiviruses, such as the feline immunodeficiency virus (FIV) (Reed et al., 2018).

A basic amino acid residue within the nucleocapsid (NC) domain of Gag is important for the recruitment of ABCE1, and Gag molecules carrying mutations in this residue fail to form fully assembled capsids (Lingappa et al., 2006). Indeed, double immunogold labeling followed by cryo-EM revealed that ABCE1 is redirected to the Gag assembly site depending on the integrity of the critical basic residue in NC (Dooher et al., 2007). Interestingly, further research showed that ABCE1 does not bind NC directly. Instead, Gag dimerization promoted by NC exposes an ABCE1-binding domain located outside the NC (Klein et al., 2011). Moreover, ABCE1 interacts with TULA (T-cell Ubiquitin Ligand) and recruits it to HIV-1 Gag assembly sites, where TULA acts in late steps of the HIV-1 replication cycle (Smirnova et al., 2008).

Evidence for the relevance of ABCE1 in HIV-1-positive patients has been reported. Resequencing analysis of the *ABCE1* gene and genomic comparisons revealed an excess of rare genetic variations in the *ABCE1* gene among HIV-1-positive African–American individuals compared to those among populations of European descent, suggesting positive selection through *ABCE1* and the surrounding genomic regions (Crawford et al., 2009; previously reviewed in Tian et al., 2012). Indeed, by using CD4^+^ T cells from healthy donors and an *ex vivo* CD4^+^ T cell HIV-1 infection system, the authors found that an insertion/deletion variant (rs9333571) in the *ABCE1* gene decreased HIV-1 permissiveness (Bleiber et al., 2005; previously reviewed in Tian et al., 2012). These findings are suggestive of ABCE1 importance in HIV-1 infection/pathogenesis. However, additional studies are necessary to correlate the findings *in vitro* regarding the role of ABCE1 in particle assembly with a possible function of this ATPase in infection *in vivo*.

#### The AAA ATPase VPS4

The ESCRT (Endosomal Sorting Complex Required for Transport) machinery comprises four distinct multimeric complexes (ESCRT-0 to ESCRT-III) that work sequentially to coordinate the formation of the ILVs of late endosomes/MVBs and the selection of ubiquitinated and non-ubiquitinated cargo proteins (daSilva et al., 2009; Dores et al., 2012a, b; Amorim et al., 2014; previously reviewed in Hurley et al., 2020). The selection of the cargo begins in subdomains of early endosomes enriched in clathrin and HRS (hepatocyte growth factor-regulated tyrosine kinase substrate), one of the components of the ESCRT-0 complex (Raiborg et al., 2002). HRS interacts with both the cargo and ESCRT-I, which also binds ESCRT-II. The ESCRT-III complex is subsequently recruited and mediates the invagination of the endosome limiting membrane and the fission of ILVs. VPS4 (vacuolar protein sorting 4), an AAA-ATPase, binds ESCRT-III subunits and catalyzes the disassembly of ESCRTs upon ATP hydrolysis. This latter process facilitates ESCRT machinery recycling and is essential for sustained ILV biogenesis (Lata et al., 2008).

In addition to ILV formation, the ESCRT machinery components function in other cellular processes requiring membrane remodeling, such as in the abscission phase of cytokinesis (Carlton and Martin-Serrano, 2007; Morita et al., 2007; Elia et al., 2011; Guizetti et al., 2011; Peel et al., 2011), and in the regeneration of the nuclear envelope during mitosis (Olmos et al., 2015, 2016; Raab et al., 2016; Ventimiglia et al., 2018; von Appen et al., 2020). HIV and other retroviruses hijack ESCRT machinery components to perform membrane fission events that are topologically equivalent to ILV biogenesis in MVBs, i.e., facing away from the cytosol (Hurley and Hanson, 2010; Peel et al., 2011; Sundquist and Kräusslich, 2012; Freed, 2015). At HIV-1 assembly sites, Gag recruits the ESCRT-I subunit TSG101 (tumor susceptibility gene 101) via the P(T/S)AP motif present in the Gag p6 domain (Huang et al., 1995; Garrus et al., 2001; Martin-Serrano et al., 2001; VerPlank et al., 2001; Demirov et al., 2002). In an alternative budding pathway, Gag recruits the ESCRT-related protein ALIX (also known AIP1 or PDCD6IP) via an YPX_*n*_L motif that is also present in the p6 domain (Strack et al., 2003; Fujii et al., 2009; Usami et al., 2009; Eekels et al., 2011). In addition to ESCRT-I and ALIX, subunits of ESCRT-II were also shown to be important for HIV-1 budding (previously reviewed in Carlson and Hurley, 2012). Notably, the EAP45 protein, a component of the ESCRT-II complex, is required for the late stages of HIV-1 replication via the YPX_*n*_L-ALIX pathway (Meng et al., 2020).

As a result of Gag interactions with ESCRT-I/ALIX and ESCRT-II, ESCRT-III and, subsequently, VPS4 are also recruited to HIV-1 assembly sites to accomplish membrane scission and viral particle release (Wollert et al., 2009; Bleck et al., 2014; Van Engelenburg et al., 2014). Interestingly, only a subset of the ESCRT-III subunits (the CHMP2 and CHMP4 families) involved in ILV formation are required for HIV-1 budding (Morita et al., 2011).

Polymerization of the CHMP4 and CHMP2 proteins (ESCRT-III subunits) is thought to drive the closure of the membrane neck of a budding virus (Hanson et al., 2008), and lead to exposure of the helical sequence domain in the CHMP2 C-terminus, which in turn binds the MIT domain of the N-terminal region of VPS4. This process allows VPS4 to remove ESCRT-III subunits from the viral membrane neck, culminating in viral membrane fission (Obita et al., 2007; Stuchell-Brereton et al., 2007; Baumgärtel et al., 2011). This VPS4 activity is coupled to ATP hydrolysis, which converts chemical energy into mechanical force to trigger the constriction and cleavage of ESCRT-III polymer ring (Monroe et al., 2017; Schöneberg et al., 2018; Maity et al., 2019). Therefore, the energy required for ESCRT-dependent HIV-1 assembly/budding comes from ATP hydrolysis mediated by VPS4 (previously reviewed in Sundquist and Kräusslich, 2012).

## HIV-1 Accessory Proteins

The HIV-1 accessory proteins [Nef, viral protein R (Vpr), viral infectivity factor (Vif), and Vpu] are not essential for virus replication *in vitro*, but they are decisive in creating an intracellular environment that allows efficient viral particle production and spread *in vivo* (previously reviewed in Sauter and Kirchhoff, 2018). In this section, we discuss how the HIV-1 accessory proteins Nef and Vpu co-opt GTPases, ATPases and their regulators and effectors to assist in virus replication and to evade host defenses.

### Negative Factor (Nef)

The HIV negative factor (Nef) protein is a critical determinant of viral pathogenesis (Deacon et al., 1995; Gulizia et al., 1997; Oelrichs et al., 1998; Learmont et al., 1999; Gorry et al., 2007). Most of Nef’s functions in infection rely on its ability to modify the trafficking of membrane proteins in host cells (previously reviewed in Pereira and daSilva, 2016; Buffalo et al., 2019). Among the targets of Nef is the ATPase ABCA1, an ABC transporter family member that mediates lipid efflux from cells and contributes to the biogenesis of HDL (high-density lipoprotein) by transferring phospholipids and cholesterol to extracellular apoA-I (apolipoproteinA-1) molecules (Santamarina-Fojo et al., 2001; Neufeld et al., 2004; Fitzgerald et al., 2010; Kang et al., 2010).

**FIGURE 3:**
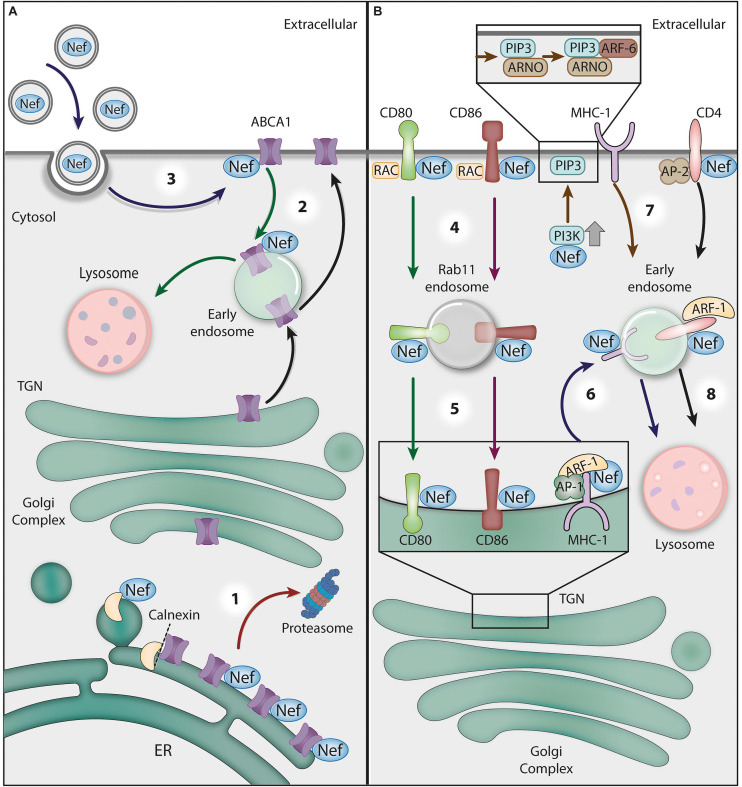
The crosstalk between HIV-1 Nef and GTPases and ATPases in protein trafficking. **(A)** The Nef-mediated downregulation of ABCA1 ATPase. (1) Nef disrupts the interaction between newly synthesized ABCA1 and the ER chaperone calnexin, leading to the targeting of ABCA1 to the ERAD/proteasomal pathway (red arrow). (2) Additionally, Nef directs ABCA1 molecules that reach the plasma membrane (PM) to lysosomes for degradation (green arrows). (3) Recently, it has been proposed that Nef obtained from extracellular vesicles taken up by bystander cells may also downregulate ABCA1 (blue arrows). **(B)** Nef hijacks GTPase activities for receptor downregulation. (4) Nef interacts with the cytosolic tails of the surface proteins CD80 and CD86 to target them for Rac1 GTPase-dependent endocytosis. Nef stimulates the translocation of Src kinase to the PM, where it activates a Rac GEF (TIAM), which in turn activates Rac1, resulting in actin polymerization nucleation sites at the cell surface. (5) After endocytosis, Nef recruits Rab11- to CD80/CD86-positive vesicles to target them to the Golgi complex (red and green arrows). (6) To prevent MHC-I delivery to the PM, Nef links the MHC-1 CT to AP-1 and induces the ARF-1 GTPase-dependent trimerization of AP-1 into an ‘open’ conformation. This promotes the recruitment of MHC-I to forming clathrin-coated vesicles destined for endosomes (dark blue arrows). (7) Alternatively, Nef activates PI3K at the PM, leading to PIP3 accumulation, which favors the recruitment of PIP3-binding ARF-6 GEF (ARNO) and the subsequent ARF-6-dependent endocytosis of MHC-I (brown arrow). (8) Nef also induces the endocytosis of CD4 via AP-2/clathrin vesicles and then targets this receptor to lysosomes for degradation through an ARF-1-dependent mechanism (black arrow).

As previously mentioned, HIV-1 assembly platforms at the cell surface are enriched in PI(4,5)P2 and lipid rafts, which contain high levels of cholesterol and sphingolipids (Nguyen and Hildreth, 2000; Ono and Freed, 2004; Hogue et al., 2011). There is evidence that Nef contributes to the enrichment of cholesterol on lipid rafts at the PM by impeding ABCA1-mediated cholesterol efflux (Zheng et al., 2003; Mujawar et al., 2006; Fitzgerald et al., 2010; Cui et al., 2012). There are at least two proposed mechanisms underlying Nef’s antagonism to ABCA1 (Figure 3). First, Nef was shown to retain ABCA1 in the ER and stimulate ABCA1 proteasome-mediated degradation (Mujawar et al., 2010). Although the binding of Nef to ABCA1 (via its C-terminal domain – ABCA1_2225__–__2231__*aa*_) was demonstrated, an ABCA1 mutant that does not interact with Nef also failed to exit the ER and was degraded in response to Nef expression (Mujawar et al., 2010). Rather than acting directly on ABCA1, Nef appears to disrupt the association between newly synthesized ABCA1 and the ER chaperone calnexin, which leads to the enhanced degradation of ABCA1 via the proteasomal/endoplasmic-reticulum-associated protein degradation (ERAD) pathway (Jennelle et al., 2014).

There is also evidence that Nef targets surface ABCA1 for lysosomal degradation (Cui et al., 2012). In support of this alternative model, the authors of one study show that in Nef-expressing cells, ABCA1 was relocated from the PM to lysosomes for degradation (Cui et al., 2012). Interestingly, the effects of Nef on ABCA1 during HIV-1 infection may not be restricted to infected cells, as exogenous Nef taken up by cells was also shown to be active in ABCA1 downregulation and cholesterol efflux reduction (Mukhamedova et al., 2019) (Figure 3). Nef antagonism of ABCA1 is likely relevant in HIV-1 pathogenesis, as ABCA1 downregulation and low HDL cholesterol levels were found in HIV-1 treatment-naïve patients, an effect that was reverted by HAART (Feeney et al., 2013; Lo et al., 2014; Thangavel et al., 2018).

Another ATPase that interacts with Nef is the vacuolar H^+^ ATPase (also known as V-ATPase or V_1_V_0_-ATPase), a multimeric complex that mediates the acidification of cellular compartments, such as the Golgi complex, endosomes and lysosomes, by pumping protons into their lumen (Oot et al., 2017). Nef interaction with V-ATPase is thought to occur via its regulatory subunit H (V1H), also known as Nef binding protein 1 (NBP1), relying on the C-terminal flexible loop of Nef (Lu et al., 1998). As the depletion of NBP1, or the disruption of NBP-1-Nef interaction, impaired the Nef-mediated reduction of cell-surface CD4 levels, the authors suggested that Nef interaction with V-ATPase plays a role in CD4 downregulation (Lu et al., 1998). Furthermore, V_1_H binds the μ2 subunit of the endocytic clathrin adaptor AP-2 (AP-2μ2), via the 133–363_*aa*_ domain of V_1_H and the N-terminal (1–145_*aa*_) domain of AP-2μ2, which led to the proposal of a refined model in which the V-ATPase connects Nef with the endocytic machinery (Geyer et al., 2002). Nef was later shown to bind AP-2 directly (Chaudhuri et al., 2007; Ren et al., 2014) and to bridge AP-2 to CD4 (Januário and daSilva, 2020; Kwon et al., 2020); therefore, the relative importance of V_1_H in this process remains an open question. Moreover, the fact that V_1_H overexpression inhibits the Nef-mediated increase in HIV-1 infectivity (Geyer et al., 2002) indicates that this host factor may be involved in additional Nef actions.

In a different role, Nef changes protein trafficking by usurping the vesicle-sorting machinery regulated by the GTPase ARF-1 (Figure 3). This strategy is applied, for instance, to prevent viral antigen presentation by major histocompatibility complex I (MHC-I) molecules, a process that contributes to HIV-1 immune evasion (Cohen et al., 1999; Specht et al., 2008; previously reviewed in Pereira and daSilva, 2016). Nef precludes the delivery of newly synthesized MHC-I molecules to the cell surface, redirecting their transport from the TGN to the endolysosomal system for degradation (previously reviewed in Pereira and daSilva, 2016). As previously mentioned, the clathrin adaptor AP-1 is recruited by ARF-1 to TGN membranes to form clathrin-coated vesicles (CCVs) that are destined for endosomes. In fact, MHC-I downregulation by Nef was shown to require AP-1 (Williams et al., 2002; Roeth et al., 2004; Lubben et al., 2007; Schaefer et al., 2008; Leonard et al., 2011; Jia et al., 2012; Tavares et al., 2020) and ARF-1 activity (Wonderlich et al., 2011). Structural analysis revealed that ARF-1-GTP binding to AP-1 triggers conformational changes in AP-1, which acquires an ‘unlocked’ state that is compatible with vesicle-cargo binding (Ren et al., 2013). Intensive research into the mechanism of MHC-I downregulation by Nef revealed that the viral protein links the cytosolic tail of MHC-I to AP-1. Specifically, in the presence of the MHC-I cytosolic tail and ARF-1, Nef induces the trimerization of unlocked AP-1 in an “open” conformation that promotes CCV biogenesis (Shen et al., 2015).

Strikingly, a very similar strategy is used by Nef to prevent the cell surface delivery of the host restriction factor BST2 (also known as Tetherin) through retention at the TGN. In the presence of the cytosolic tail of BST2, Nef changes ARF-1-mediated AP-1 trimerization to a “closed” conformation that appears to be incompatible with CCV assembly (Shen et al., 2015). The structural basis for the cargo-dependent diversity of Nef-induced AP-1 trimerization was recently elucidated, and it was also revealed that it is finely tuned by the phosphorylation state of Nef (daSilva and Mardones, 2018; Morris et al., 2018).

The hijacking of ARF-1-mediated trafficking pathways by Nef may also occur beyond the TGN. It has been reported that ARF-1 activity stabilizes Nef in endosome membranes and may facilitate Nef-mediated targeting of CD4 to this compartment for transport to lysosomes for degradation (Fauré et al., 2004). In fact, the expression of an ARF-1 dominant-negative form inhibited Nef-CD4 complex migration toward the lysosome (Fauré et al., 2004), highlighting the importance of ARF-1 in other receptor downregulation strategies utilized by Nef. Interestingly, it has been shown that Nef uses a variant of AP-1 (AP-1-γ2) to retain CD4 (Tavares et al., 2017) and MHC-I (Tavares et al., 2020) in endosomes for lysosomal delivery.

Nef-induced endocytosis via ARF-6 was also proposed to contribute to MHC-I downregulation. Specifically, Nef was shown to activate class I phosphatidylinositol 3-kinases (PI3K), leading to the accumulation of phosphatidylinositol (3,4,5)-triphosphate [PI(3,4,5)P3] at the inner leaflet of the PM. This was proposed to favor PM recruitment of the PI3P-binding protein ARF nucleotide-binding site opener (ARNO), an ARF-6 GEF, which stimulates the ARF-6-dependent endocytosis of MHC-I (Blagoveshchenskaya et al., 2002; Hung et al., 2007). Indeed, the pharmacological inhibition of class I PI3K or the overexpression of inactive ARNO was shown to compromise efficient MHC-I downregulation by Nef (Blagoveshchenskaya et al., 2002; Hung et al., 2007).

In addition to MHC-I, Nef also removes the immune costimulatory molecules CD80 and CD86 from the surface in antigen-presenting cells (Figure 3), such as DCs and macrophages, thus interfering with T cell priming (Chaudhry et al., 2007, 2008). The mechanism differs from that of MHC-I downregulation and involves the activity of Rac1, a small GTPase within the Rho family mentioned earlier in this review. Nef was shown to promote the translocation of Src kinase to the cell periphery, where Src promotes Rac GEF (TIAM) activation, which in turn activates Rac1 (Chaudhry et al., 2007). At the cell surface, Nef interacts with the CD80 and CD86 cytosolic tails and targets these molecules to actin polymerization nucleation sites at the PM that support endocytosis, possibly via Nef co-interaction with Rac1 itself, facilitating CD80/CD86 internalization (Chaudhry et al., 2007). Additionally, Nef was proposed to recruit Rab11 to vesicles containing internalized CD80/CD86 to return them to the Golgi complex (Chaudhry et al., 2008) (Figure 3).

### Viral Protein U (Vpu)

The viral protein U (Vpu) encoded by the HIV-1 genome, is a type I transmembrane protein that contributes to viral immune evasion by antagonizing host proteins that are detrimental to virus replication and dissemination. Vpu acts by modifying the intracellular distribution of its targets, frequently directing them to a degradative pathway. Among the targets of Vpu are CD4, HLA-C, Tim-3, BST2 (Tetherin), CD1d and NTB-A (Strebel, 2014; previously reviewed in Sauter and Kirchhoff, 2018; Prévost et al., 2020). In many cases, the mechanisms underlying the actions of Vpu depend on the participation of GTPases.

Among the most intensively studied functions of Vpu is its antagonism to BST2, an interferon-induced host restriction factor that attenuates viral transmission by impairing the release of HIV-1 and other enveloped viruses from infected cells (Neil et al., 2008; Van Damme et al., 2008; Perez-Caballero et al., 2009). Vpu is thought to remove BST2 from HIV budding sites at the PM mainly by preventing naturally internalized and newly synthesized BST2 molecules from reaching the cell surface, eventually leading to its downregulation (Schmidt et al., 2011). Similar to the downregulation of MHC-I by Nef, Vpu also hijacks the ARF-1/AP-1 sorting machinery to antagonize BST2. Specifically, Vpu was shown to form a tripartite complex with BST2 and AP-1 at the TGN that is thought to load BST2 into CCVs destined for endosomes and to block the resupply of BST2 to the PM (Jia et al., 2012).

Vpu was also shown to target BST2 to lysosomes for degradation (Mitchell et al., 2009; Dubé et al., 2010). In this regard, the GTPase Rab7A plays a key role in the constitutive turnover of BST2, and its depletion was shown to compromise BST2 endolysosomal degradation induced by Vpu (Caillet et al., 2011). HRS, a component of the ESCRT-0 machinery, has also been shown to participate in Vpu-mediated BST2 downregulation by recognizing ubiquitinated BST2 and targeting it for lysosomal degradation via the MVB pathway (Janvier et al., 2011). Therefore, another energy-harnessing molecule likely involved in the antagonism of BST2 by Vpu is the AAA-ATPase VPS4, whose activity, as previously discussed, is essential for ESCRT function at MVBs.

## Final Considerations and Future Directions

The spatiotemporal control of viral and host proteins distribution in infected cells is key to several steps in the HIV-1 replication cycle. These processes require specialized transport machinery and demand chemical energy, which is supplied by the host cells through ATP and GTP hydrolysis. With the exception of the reverse transcriptase and the integrase, other HIV-1 proteins do not possess known ATP or GTP binding properties and are incapable of directly harnessing energy from these molecules. Instead, HIV-1 factors repurpose host GTPases (such as Dynamin and small GTPases – Ran, Rab, ARF, and Rho family members) and ATPases (such as ABCE1, ABCA1 and VPS4) to regulate: (1) the subcellular distribution of viral components, (2) the subcellular distribution of host proteins that affect virus replication and infectivity and, (3) membrane remodeling reactions required for viral entry and assembly/egress. Several examples of these strategies are discussed here, and many others are likely to arrive.

Despite the outstanding effort in understating the interplay between HIV-1 and energy-related proteins, questions issues remain unsolved. These include, but are not limited to: (1) Why the function of Dynamin in endocytosis, Rab5, and Rab7 is relevant in HIV-1 entry in only some cell types? (2) Which is the precise role of ABCE1 in HIV-1 infection *in vivo*? (3) What are the mechanisms used by the HIV-1 to activate Rho GTPases and their downstream effectors to modulate the actin cytoskeleton? (4) How can ARF-1, Rab8A, and Rab29-mediated transport pathways influence Gag targeting tetraspanin-enriched microdomains during the VSs? (5) Which is the primary model that explains ABCA1 downregulation by Nef? (6) Why is the binding of Nef to the vacuolar H+ ATPase important in CD4 downregulation if Nef can directly bridge CD4 to AP-2?

To address these questions, efforts should be directed to using cellular models that are physiologically relevant for HIV infection and validating the findings using primary human cells and animal models when possible. RNAi and knockout library screening techniques, the several novel high-resolution imaging approaches, and the multiple structural and biochemical methods that became available in recent years will help shed light on these unsolved issues.

Besides contributing to viral fitness, deviating these energy-related molecules from their normal cell function may have broader cellular physiology effects, which likely influences HIV-1 pathogenesis. In this respect, drugs that interfere with specific GTPases or ATPases function may represent potential new anti-HIV agents candidates. Moreover, efforts to discover specific interactions between HIV-1 factors and energy-related molecules may offer new targets for small molecule inhibitors to develop additional anti-HIV therapies.

## Author Contributions

All authors listed have made a substantial, direct and intellectual contribution to the work, and approved it for publication.

## Conflict of Interest

The authors declare that the research was conducted in the absence of any commercial or financial relationships that could be construed as a potential conflict of interest.

## Abbreviations

HIV: human immunodeficiency virus
SIV: simian immunodeficiency virus
FIV: feline immunodeficiency virus
CCR5: C–C chemokine receptor type 5
CXCR4: C-X -C chemokine receptor type 4
AIDS: acquired immunodeficiency syndrome
HAART: highly active antiretroviral therapy
PIC: pre-integration complex
PM: plasma membrane
GDP: guanosine diphosphate
GTP: guanosine triphosfate
ATP: adenosine triphosphate
GAPs: GTPase-activating proteins
GEFs: guanine nucleotide exchange factors
ABC: ATP-binding cassette
Env: HIV envelope glycoprotein (precursor gp160, surface gp120, and transmembrane/cytosolic gp41)
Gp41C: cytosolic tail of gp41
Nef: negative factor
Gag: group-specific antigen
NC: nucleocapsid domain of Gag
MA: matrix domain of Gag
Pol: polymerase
Tat: *trans*-activator of transcription
Vpu: viral Protein U
Vif: viral infectivity factor
Vpr: viral protein R
Rev: regulator of virion expression
RREs: rev response elements
CME: clathrin-mediated endocytosis
Ran: Ras-related nuclear
TRN-SR2/TNPO3: transportin-SR2
VLPs: virus-like particles
ARFs: ADP-ribosylation factors
GGA: golgi-localized, γ-ear containing, ARF-binding
AP: adaptor protein complex
RNAi: RNA interference
Rab: Ras-related in brain
BST2/Tetherin: bone marrow stromal cell antigen 2
DCs: dendritic cells
VSs: virological synapses
TGN: *trans*-Golgi network
ER: endoplasmic reticulum
MPRs: mannose 6-phosphate receptors
TIP47: tail-interacting protein of 47 kDa
ABCE1: ATP-binding cassette sub-family E member 1
TULA: T-cell ubiquitin ligand
FIP1C: Rab11-family interacting protein 1C
PI(4)P: phosphatidylinositol 4-phosphate
PI(4,5)P2: phosphatidylinositol (4,5)-bisphosphate
PI(3,4,5)P3: phosphatidylinositol (3,4,5)-triphosphate
PI4KIIα: phosphatidylinositol 4-kinase type 2 α
Cdc42: cell division control protein 42 homolog
Rac1: Ras-related C3 botulinum toxin substrate 1
RhoA: Ras homolog family member A
DC-SIGN: dendritic cell-specific ICAM-3-grabbing non-integrin
LARG: leukemia-associated Rho guanine nucleotide exchange factor (Rho GEF)
Tiam1: T-lymphoma invasion and metastasis-inducing protein 1 (Rac GEF)
ESCRT: endosomal sorting complex required for transport
HRS: hepatocyte growth factor-regulated tyrosine kinase substrate
VPS4: vacuolar protein sorting 4
ILVs: intraluminal vesicles
MVBs: multivesicular bodies
TSG101: tumor susceptibility gene 101
ALIX/AIP1/PDCD6IP: programmed cell death 6-interacting protein
CHMP: charged multivesicular body protein
ABCA1: phospholipid-transporting ATPase ABCA1
HDL: high-density lipoprotein
apoA-I: apolipoproteinA-1
ERAD: endoplasmic-reticulum-associated protein degradation
CCV: clathrin-coated vesicle
MHC-I: major histocompatibility complex I
PI3K: class I phosphatidylinositol 3-kinase
ARNO: ARF nucleotide-binding site opener.
